# Trichinosis

**Published:** 1874-11

**Authors:** 


### TRICHINOSIS.

Small and frequently repeated doses of oil of turpentine, to the extent of not causing diarrhœa or other unfavorable symptoms, is believed to be efficacious in a very short time in destroying every worm in the body, as it is very volatile, and soon pervades the entire flesh.

